# Hemagglutinin stem reactive antibody response in individuals immunized with a seasonal influenza trivalent vaccine

**DOI:** 10.1007/s13238-015-0160-6

**Published:** 2015-05-06

**Authors:** Xiaopeng Zhao, Kun Qin, Jinlei Guo, Donghong Wang, Zi Li, Wenfei Zhu, Liqi Liu, Dayan Wang, Yuelong Shu, Jianfang Zhou

**Affiliations:** National Institute for Viral Disease Control and Prevention, Chinese Center for Disease Control and Prevention, Key Laboratory for Medical Virology, National Health and Family Planning Commission, Beijing, 102206 China

**Dear Editor,**

Influenza is a contagious, acute respiratory disease caused by influenza viruses. Type A influenza virus has a broad host range and has caused substantial human morbidity and mortality. There are sixteen subtypes of influenza A virus so far, which are divided into two distinct groups: group 1 (H1, H2, H5, H6, H8, H9, H11, H12, H13 and H16) and group 2 (H3, H4, H7, H10, H14 and H15) (Corti et al., 2011). Seasonal H1 and H3 subtype influenza viruses are circulating in the human population. Vaccination is widely used for the prophylaxis and the antibodies (Abs) induced by the vaccine were conventionally thought to be mainly directed against the variable head region in HA. Those Abs could block viral binding to the sialic acid receptors on cell surface (Skehel and Wiley, 2000). They are typically effective against antigenically close-related strains and thus need to be updated annually.

Recently, a number of broadly neutralizing monoclonal antibodies (BnAbs) directed against the HA stem have been identified, including CR6261, F10, FI6v3, FE43 and FE17 and so on (Corti et al., 2010; Corti et al., 2011; Sui et al., 2009; Throsby et al., 2008). The stem-reactive Abs also performed neutralizing activity by either blocking viral fusion or preventing the cleavage by host protease (Ekiert et al., 2009). Furthermore, the development of those BnAbs is not only correlated with antigen immunogenicity but also with the host genetic background (Ellebedy et al., 2014; Li et al., 2012; Pappas et al., 2014). However, whether human serum has such kind of Abs and the frequency or magnitude of the Abs in peripheral blood are not fully answered. BnAbs against Group 1 HA have been discovered from 4 out of 24 donors vaccinated by seasonal-flu vaccine (Corti et al., 2010). Here, we focused on the Chinese adults vaccinated by a seasonal trivalent vaccine in 2009 containing A/Brisbane/59/2007 (Br59, H1N1), A/Brisbane/10/2007 (H3N2), and B/Florida/4/2006. The serum Ab profile on Day 0 and Day 21 from 49 volunteers was determined.

Initially, the Ab response to homologous Br59-HA was measured by enzyme-linked immunosorbent assay (ELISA). Most donors had detectable Br59-HA binding antibody titer before vaccination and the geometric mean titer (GMT) was 105.93 ± 1.17. As expected, the strain-specific Ab titer increased dramatically after vaccination (*P* < 0.0001) and the GMT was up to 772.68 ± 1.18 (Fig. 1A). In consistent with the Hemagglutination-inhibition (HI) titers reported in the previous study (Wu et al., 2011), a more than 2.5-fold increase of Br59-HA binding Ab titer was detected in 87.8% of the cohort after immunization (Fig. 1B), suggesting that the vaccine was efficient to induce homologous H1 subtype Ab response.

**Figure 1:**
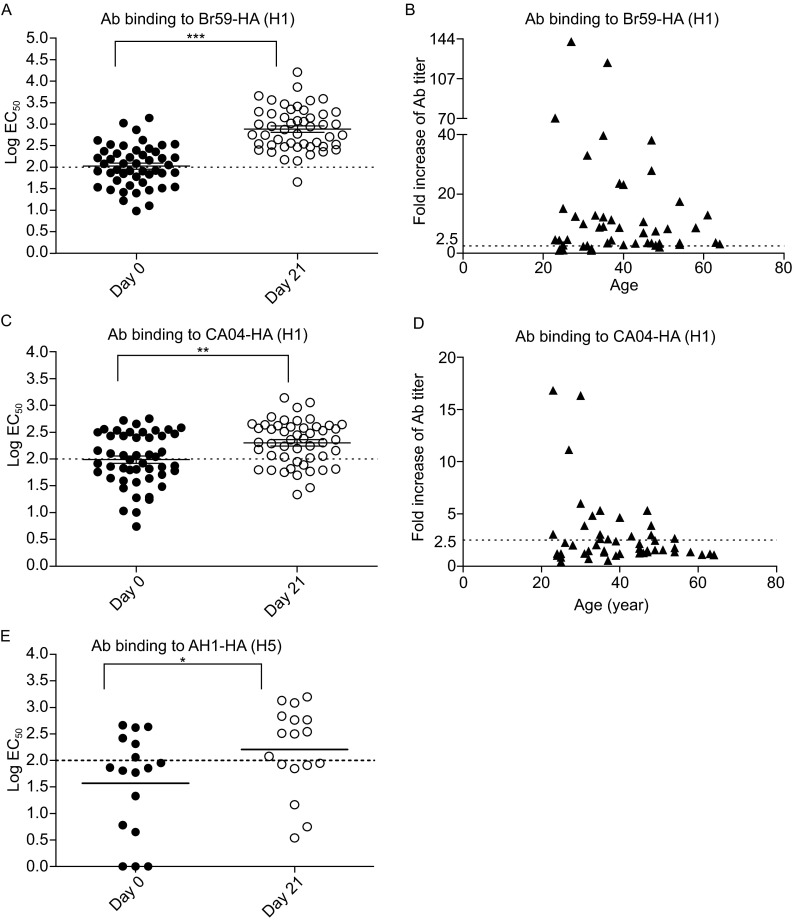
The level of serum Ab binding to homologous Br59-HA and cross-reactivity of serum Ab to 2009pdm H1 and H5. (A, C and E) Binding Ab titer of Br59-HA (A), CA04-HA (C), AH1-HA (E) before and after seasonal influenza vaccination. The solid round (●) represents serum Ab titer on Day 0 and the empty round (○) represents the Ab titer on Day 21 after vaccination. Levels of serum Ab bound to immobilized HA were detected by ELISA, as described in Supplementary material. The half maximal (50%) effective concentration (EC_50_) of HA-binding Ab was calculated in a constrained non-linear regression (curve fit) analysis assay using GraphPad Prism 5. The dashed line represents the lowest dilution of tested sera. Data are expressed as geometric mean ± standard error mean (GEM ± SEM) of the vaccinees. *P < 0.05, **P < 0.01, ***P < 0.0001. (B and D). The titer increase-fold of Abs to Br59-HA (B) and CA04-HA (D) from the 49 vaccinees pre- and post-vaccination. The solid triangle (▲) represents the fold increase of HA binding Ab titer. The dashed line denotes an incease fold of 2.5

To determine the cross-reactive Abs after vaccination, we then screened the 49 paired sera using recombinant HA derived from A/California/04/2009 (CA04, H1N1). As shown in the Fig. 1C, a low level of CA04-HA binding Abs, around 97.27 ± 1.18 was observed, the Ab response was boosted after vaccination and the GMT was 200.45 ± 1.14. Among them, 17 donors had a more than 2.5-fold increase of CA04-HA binding Ab titer after immunization (Fig. 1D). We further measured if there were any heterosubtypic Abs against H5-HA in the 17 paired sera. Among them, only 6 had a detectable H5-HA binding Abs and the GMT was 37.24 ± 1.69 on Day 0, other 11 had a very low, even undetectable titer. While after vaccination, a 2.5-fold increase of H5-HA binding Ab titer was found in 11 donors and the GMT was 160.32 ± 1.56 (*P* < 0.05, Fig. 1E).

Since Br59-HA has great antigenic variability with CA04-HA in the head region, with the amino acid identity in the stem domain being high as 89.5%, we speculated the cross-reactivity of the Abs might target HA stem, a highly conserved domain in HA (Table S1). As stem-reactive Abs were present at an extremely lower level and any biological interference caused by agents from sera disturbing the cross-binding Abs remained uncertain, we enriched the immunoglobulins (Igs) in the 17 paired sera by Protein A and measured their neutralizing activity with pseudovirus particles (pps) bearing a chimeric HA (cH5/1 SZ) as described in the Materials and Methods. The neutralizing Ab titer was defined as the reciprocal of the serum dilution causing a 90% reduction of relative luciferase unite (RLU) compared to the control. Seven of them showed neutralizing activity, four had a titer of more than 8 after immunization, and one with a titer of 2 and two had a titer of 2 pre- and post-immunization (Fig. 2A). The correlation analysis between Br59-HA binding Abs and the neutralizing Abs in the 7 donors revealed that the cross-reactive neutralizing antibodies were induced by vaccination (*R*^2^ = 0.7659, *P* = 0.0014) (Fig. 2B).

**Figure 2:**
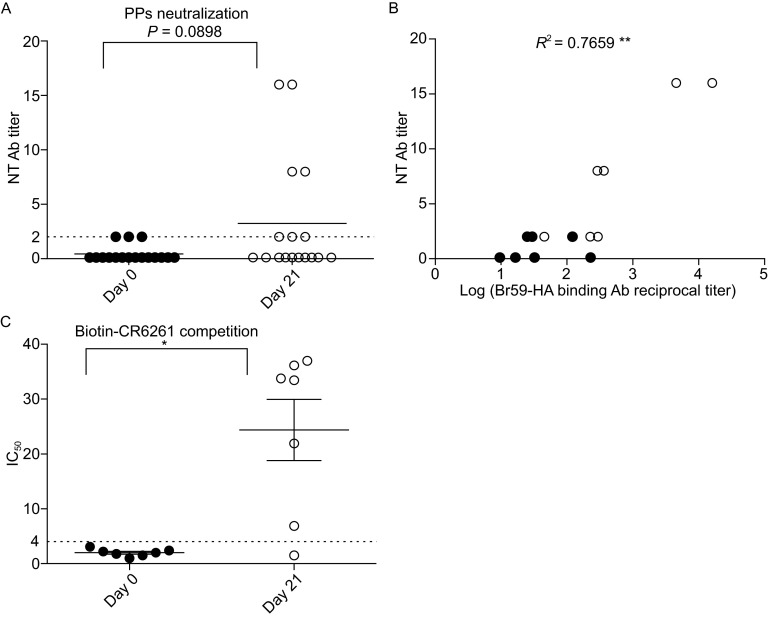
Cross-neutralizing Ab response to cH5/1 pps and HA stem-reactive CR6261-like antibody response in the selected volunteers. (A) The cH5/1 pps neutralizing Ab titer of the 17 donors with cross-binding activity to 2009pdm H1. The neutralizing (NT) Ab titer was defined as the reciprocal of the serum dilution causing a 90% reduction of relative luciferase unite (RLU) compared to the control. (B) Correlation between the Br59-HA binding titer and cH5/1 pps neutralizing antibody titer. Correlation analysis was tested using Nonparametric correlation (Spearman). **P < 0.01. (C) The CR6261-like antibody titers of the 7 paired sera possessing pps neutralizing activity were detected with competitive ELISA as described in Supplementary material. The the half maximal (50%) inhibitory concentration (IC_50_) of the sera was calculated in a non-linear regression (curve fit) analysis assay using GraphPad Prism 5 or two-point calculation method. *P < 0.05. The dashed line represents the detection threshold. The solid round (●) represents serum Ab titer on Day 0 and the empty round (○) represents the Ab titer on Day 21 after vaccination

To define the antigen region recognized by the cross-reactive Abs, we used a competitive ELISA by mixing the serial-diluted Igs from the same volume (400 microliter, µL) of sera with a well-defined HA stem mAb CR6261, which has broadly neutralizing epitope crossing Group 1 HA subtype viruses (Ekiert et al., 2009). As shown in the Fig. 2C, there was undetectable CR6261-like Ab level in the pre-immunization sera of the 7 donors. After vaccination, all but one individual had a remarkable increase of CR6261-like Ab titer (*P* = 0.0018). The lowest detection threshold of Ig purified from 400 µL sera was 180 µg/mL (equivalent to 9 µg Ig/well). Approximately, 0.02%–0.13% CR6261-like Ab was present in total Ig after vaccination. The results indicated that the seasonal influenza vaccination did induce stem-reactive antibodies.

The Ab response of the 7 donors could be grouped into three categories by HI titers, the group of low/non response, who had a $\leqslant \!4{\text{-fold}}$ HI titer increase; moderate, ranging from 4- to 100-fold increase and the robust response with an increase of above 100-fold. However, in combination with the assays for cross-reactive Ab responses, we found the welÌl-established serological standard could not comprehensively evaluate the humoral response after influenza vaccination. We observed that Sample 072 and 117, presumably belonging to low/non response group did possess the cross-reactive Abs, including CR6261-like stem (1:33.78, 1:33.45) and the HA head of A/California/07/2009 reactive Abs (1:160) (Tables S2 and S3). Comprehensive serological profiling in our cohort indicated that only the HI titer cannot fairly assess the vaccine potency in humans, thus the protective cross-reactive Abs should be employed for vaccine evaluation in future.

Generally, in our cohort, 17 showed the cross-reactivity to 2009pdm H1 and 11 out of 17 exhibited cross-reactivity to H5. In parallel, we observed 7 out of the 17 had the neutralizing activity to cH5/1(SZ) pps. Six (12.24%) possessed CR6261-like Abs recognizing HA stem. In consistent with the study by Corti et al., our data suggested that seasonal vaccination could induce HA stem-directed Abs with cross-neutralizing effects on H5N1 pps. Moreover, our pre-immunization serum samples also had basal heterosubtypic HA-binding and cross-neutralizing activity as previous studies (Corti et al., 2010; Sui et al., 2011). In contrast to Sui’s report, 0.001% of HA-stem reactive Abs by competing with F10, sharing the same epitope as that of mAb CR6261, was present in the intravenous immunological globin at a concentration of 100 mg/mL pre-vaccination (Ekiert et al., 2009; Sui et al., 2009), we only detected the rise of CR6261-like Abs on Day 21 in our cohort and the fraction is around 0.02%–0.13% in the total purified Igs with an average concentration of 10 mg/mL. Of note, we discovered that varied extent or width of the cross-reactivity existed although some vaccinees showed a low or non-response to HA head of homologous vaccine strain. The differential HA-specific and HA cross-reactive response in individuals may be resulted from host genetic and background immunity (Pappas et al., 2014). As many cross neutralizing Abs directing to NA, M2 or epitopes located at HA head have been documented (Doyle et al., 2013; Wei et al., 2011; Whittle et al., 2011), further investigations on cross-reactive Abs profiling should be performed.

## Electronic supplementary material

Supplementary material 1 (PDF 148 kb)
