# Supplementary material for: Hemagglutinin stem reactive antibody response in individuals immunized with a seasonal influenza trivalent vaccine
Source: Protein Cell. 2015 May 6;6(6):453–7. doi: 10.1007/s13238-015-0160-6 (PMC4444808; doi:10.1007/s13238-015-0160-6)
Supplement: Supplementary file 1 — Supplementary material 1 (PDF 148 kb) [file 13238_2015_160_MOESM1_ESM.pdf]

Supporting Online Materials for

Title

Hemagglutinin Stem Reactive Antibody Response in Individuals Immunized with a Seasonal Influenza Trivalent Vaccine

Running Title

Anti-HA stem Ab produced in the seasonal flu vaccinees.

Keywords: influenza virus, vaccine, hemagglutinin (HA), cross-reactive antibody

Authors and Affiliations

Xiaopeng Zhao[1*], Kun Qin[1*], Jinlei Guo[1], Donghong Wang[1], Zi Li[1], Wenfei Zhu[1], Liqi Liu[1], Dayan Wang[1], Yuelong Shu[1], Jianfang Zhou[1†]

1 National Institute for Viral Disease Control and Prevention, Chinese Center for Disease Control and Prevention, Key Laboratory for Medical Virology, National Health and Family Planning Commission, Beijing, 102206, PR. China.

* These authors contributed equally in this study.

†Correspondence footnote:

Dr. Jianfang Zhou, National Institute for Viral Disease Control and Prevention, Chinese Center for Disease Control and Prevention, Key Laboratory for Medical Virology, National Health and Family Planning Commission. 155 Changbai Road, Beijing, 102206, PR. China. Tel: 86-010-63580764, Fax: 86-010-63580764. E-mail: jfz@cnic.org.cn

This file includes:

Materials and Methods

Reference

Table S1

Table S2

Table S3

Materials and Methods

Human serum samples

The serum samples were obtained from a previous study (Wu et al., 2011)and were stored at the -40°C till use. Our cohort was individuals who received a trivalent vaccine (2009-2010) at Day 0 and the serum samples collected at Day 0 and Day 21 from 49 volunteers were used in the study. The 2008 vaccine contained A/Brisbane/59/2007(H1N1), A/Brisbane/10/2007 (H3N2), and B/Florida/4/2006. The protocol in the study was approved by the ethics review committee of National Institute for Viral Disease Control and Prevention, China CDC.

Cells and viruses

Madin-Darby canine kidney (MDCK) cells and human kidney 293T cells were obtained from the American Type Culture Collection (ATCC, USA). The MDCK cells were cultured in minimal essential medium (MEM, Gibco, Grand Island, NY) supplemented with 10% fetal bovine serum (FBS) and 1×glutamine (Gln); the 293T cells were cultured in Dulbecco's minimal essential medium (DMEM, Gibco, Grand Island, NY) supplemented with 10% FBS, 1×Gln and 25mM HEPES. The reverse genetic derived cH5/1 virus with the chimeric HA gene (the head region from A/Bar-headed Goose/Qinghai/1/05(H5N1) and the stem region from PR8) and other seven gene segments from A/California/04/2009(H1N1), namely cH5/1(QH) virus, was grown in 9-day-old embryonated eggs. Then the allantoic fluid was collected and stored at -70°C until use.

Enzyme-linked immunosorbent assay (ELISA) detecting the HA-binding

antibodies

The serum samples for ELISA testing were pre-treated at 56°C for 30min. The three subtypes of recombinant HA protein was purchased from Sino Biological Inc (Beijing, China). Briefly, the 96-well plates (Costar-Corning, USA) were coated with 100ng per well of HA protein diluted in coating buffer (Kirkegaard & Perry Laboratories, USA) overnight at 4°C. The plates were blocked for 2h at 37°C with 0.05% Tween-20 PBS (PBST) containing 5% skim milk. After washing, serially diluted serum samples were applied to the HA-coated plates and incubated at 37°C for 1h. Then the plates were washed 6 times with PBST, secondary rabbit antihuman IgG-horseradish peroxidase (Sino Biological Inc, China)(1:10000 dilution) was added and incubated for 1 hour at 37°C followed by addition of tetramethylbenzidine substrate solution (TMB, BD, USA), the reaction was stopped by 2M H_2SO_4 and optical density(OD) were measured at 450 nm. The half maximal (50%) effective concentration (EC_{50}) of HA-binding Ab was calculated in a constrained non-linear regression (curve fit) analysis assay by using GraphPad Prism 5 (GraphPad Software Inc).

Preparation of cH5/1 influenza virus pps

The influenza virus pps were produced using a retrovirus-based pseudotype system via the expression of influenza virus HA, NA, Gag-Pol, and the luciferase reporter gene in a 293T cell expression system as previous reports (Du et al., 2010). Briefly, 1.2×10^6 293T cells were co-transfected with three plasmids using TurboFect Transfection Reagent (Thermo Scientific, USA): 2μg lentivirus vector pNL4-3.luc containing a luciferase reporter gene, 0.5μg plasmid expressing chimeric cH5/1 HA

(SZ) and 1μg plasmid encoding NA gene from A/Guangxi/01/2008(H5N1). After overnight incubation, cells were washed with DMEM and cultured in DMEM containing 2.5% FBS and 25mM HEPES. For additional 48hs, the supernatant was harvested and centrifuged at 2500rpm/min to remove the cell debris, and the pps were collected from the supernatants by filtration through a 0.45μm Durapore polyvinylidene difluoride (PVDF) membrane filter (Millipore, Ireland). The supernatant containing pps was stored at 4°C and used within 1 month for further neutralization assay.

Serum immunoglobulin (Ig) G purification and enrichment

The IgG in the selected paired sera were purified by Protein A HP Spin Trap (GE Healthcare, USA) according to the manufacturer's instruction. Four hundred micro liter sera from each sample was used for downstream processing. Briefly, two-fold diluted sera were added into protein Spin A and incubated for 4 minutes while gently mixing at room temperature. The columns bound with IgG were washed two times with 600μL binding buffer (0.05M Tris, 0.15M NaCl, pH 7.5) and the IgG was eluted into a microcentrifuge containing 12μL neutralizing buffer (1M Tris-HCl, pH 9.0) by centrifuging for 30s at 2000rpm/min after addition of 200μL elution buffer (0.1M Glycine-HCl, pH 2.9).

Pseudovirus titration and neutralization assay

The 96-well cell culture plate was seeded with 3×10^4 MDCK cells/well and cultured at 37°C overnight. Then the monolayer MDCK cells were washed twice with PBS and incubated with serially diluted influenza cH5/1(SZ) pps in the presence of 2μg/mL

N-tosyl-L-phenylalanine chloromethyl ketone (TPCK)–treated trypsin (Sigma, USA) at 37°C for 48h. After 48h incubation, cells were lysed and luciferase activity was measured by a Luciferase assay system (Promega, USA) according to the manufacturer's instruction. The relative luciferase unite (RLU) of pps around $2\sim9\times10^3$ was used for neutralization test. Fifty-five microliter two-fold serially diluted IgG was incubated with equal volume of pps for 1h, then the mixture (100μL) was added to the 96-well plate which was seeded with monolayer MDCK cells and incubated for 48h at 37°C. The neutralizing antibody titer was defined as the reciprocal of the serum dilution causing a 90% reduction of RLU compared to the control.

Hemagglutination-inhibition (HI) assays

HI assay was performed as described (Influenza, 2011). Briefly, purified IgG from paired serum samples were two-fold diluted in the V-bottom plates, followed by addition of equal volume of reassortant cH5/1 (QH) virus, Br59 or A/California/07/2009(H1N1) (CA07) virus which had 4 HAU/25μL and incubated at room temperature (RT) for 30min. Then 50μL 1% (vol/vol) turkey red blood cells were added and incubated for 30min at RT. The HI titer was identified as the highest dilution that prevented hemagglutination of red blood cells.

Competitive ELISA detecting stem-reactive antibodies

The CR6261 MAb was transiently expressed according to the VH/L sequence (accession number: HI919029/HI919031) and purified using Protein A. Then the antibody was biotinylated with Biotinamidocaproate N-hydroxysuccinimide ester (BNHS) (Sigma, USA) according to the manufacturer's instructions. We performed

ELISA with CA04-HA to detect the binding ability of various concentrations of Biotin-CR6261. Competition ELISA was conducted as previous report with minor modification(Sui et al., 2011): The 96-well plates were coated with 100ng CA04-HA protein per well overnight at 4°C and then were blocked with PBST containing 5% skim milk for 2h at 37°C. The purified IgG of serum samples were two-fold diluted and mixed with equal volume of biotinylated CR6261 (240ng/mL, 12ng CR6261 per well), then the mixture was added into the 96-well plates coated with CA04-HA protein and incubated at 37°C for 1h. After washing, 100μL strepavidin-HRP (R&D, USA)(1:200 diluted in PBS) was added into the plates. After incubation at 37°C for 1h, 100μL TMB was added and the reaction was stopped by addition of 2M H_2SO4. The OD was measured at 450nm and the half maximal (50%) inhibitory concentration (IC_{50}) of the sera were calculated in a non-linear regression (curve fit) analysis assay by using GraphPad Prism 5 or two-point calculation method.

Statistical analysis

Differences between groups were tested using non-parametric Mann–Whitney analysis of t test by GraphPad Prism 5; correlation analysis was tested using non-parametric correlation (Spearman) by GraphPad Prism 5. $p<0.05$ was considered statistically significant.

Reference

Du, N., Zhou, J., Lin, X., Zhang, Y., Yang, X., Wang, Y., and Shu, Y. (2010). Differential activation of NK cells by influenza A pseudotype H5N1 and 1918 and 2009 pandemic H1N1 viruses. Journal of virology 84, 7822-7831.

Influenza, W.G. (2011). Manual for the laboratory diagnosis and virological surveillance of influenza.

Sui, J., Sheehan, J., Hwang, W.C., Bankston, L.A., Burchett, S.K., Huang, C.Y., Liddington, R.C., Beigel, J.H., and Marasco, W.A. (2011). Wide prevalence of heterosubtypic broadly neutralizing human anti-influenza A antibodies. Clinical infectious diseases : an official publication of the Infectious Diseases Society of America 52, 1003-1009.

Wu, J., Zhong, X., Li, C.K., Zhou, J.F., Lu, M., Huang, K.Y., Dong, M., Liu, Y., Luo, F.J., Du, N., *et al.* (2011). Optimal vaccination strategies for 2009 pandemic H1N1 and seasonal influenza vaccines in humans. Vaccine 29, 1009-1016.

Table 1 Amino acid identity of the HA head and stem domain

Virus name	HA domain	CA04	AH1	PR8
Br59	HA head	67.3%	45.0%	77.1%
CA04	HA head		43.7%	70.8%
AH1	HA head			47.6%
Br59	HA stem	89.5%	75.8%	94.6%
CA04	HA stem		77.4%	90.1%
AH1	HA stem			76.1%

The amino acid sequences were downloaded from NCBI and the amino acid identity among different subtype HAs was analyzed by the BioEdit software. The amino acid between the Cys52 and Cys277 (H3 numbering) was the head region of HA, the remained region of HA was the stem region of HA molecular.

Table 2 HI titer of the sera with neutralizing activity to cH5/1 pps

Sample No.	cH5/1(QH)		Br59		CA07	
	Day 0	Day 21	Day 0	Day 21	Day 0	Day 21
S010	<1	<1	<10	80	<10	40
S048	<1	<1	<10	160	<10	<10
S063	<1	<1	<10	1280	<10	<10
S072	<1	<1	<10	20	<10	160
S078	<1	<1	<10	80	<10	<10
S117	<1	<1	80	80	<10	<10
S139	<1	<1	<10	40	<10	<10

The purified IgG from 7 paired serum samples with neutralizing activity were selected for HI assay with A/Brisbane/59/2007(H1N1, Br59), A/California/07/2009(H1N1, CA07) and a reassortant cH5/1(QH) virus.

Table 3 HA head-specific and cross-reactive response in the sera with neutralizing activity

Category	Sample ID	Fold increase			
		Br59 HI	CA07 HI	pps NT	CR6261-like
Low or non	S117	1	Nil	4	16.7
	S072	4	32	4	16.8
Moderate	S139	8	Nil	1	Nil
	S010	16	8	1	3.4
	S078	16	Nil	16	11
	S048	32	Nil	32	18.2
Robust	S063	256	Nil	32	18.4

The purified IgG from 7 paired serum samples possessing pps neutralizing activity are grouped into three categories according to the fold increase of HI titer against homologous Br59 virus. Low or non denotes the fold increase less than or equal to 4; Moderate denotes that seroconversion factor ranged from 4 to 100; Robust represents a seroconversion factor which is above 100. Nil represents an undetectable HI titer or CR6261-like antibody level in the serum.
